# Local Anesthetics in Diabetic Retinopathy Procedures: A Comprehensive Review With a Focus on Lidocaine-Based Pain Control

**DOI:** 10.7759/cureus.87632

**Published:** 2025-07-09

**Authors:** Kimia Rezaei, Parsa Riazi Esfahani, Mina Balen, Tri Brian Nguyen, Victoria P Farasat, Akshay J Reddy, Shazia Sheikh

**Affiliations:** 1 Medicine, University of California Riverside School of Medicine, Riverside, USA; 2 Medicine, California University of Science and Medicine, Colton, USA; 3 Physiology, University of California Los Angeles, Los Angeles, USA

**Keywords:** anesthesia, diabetic retinopathy, lidocaine, pain management, panretinal photocoagulation, surgical outcomes

## Abstract

This review investigates commonly used local anesthetic agents and administration methods in diabetic retinopathy (DR) procedures, such as pan-retinal photocoagulation, intravitreal injections, and vitrectomy, focusing on pain control and procedural outcomes. A systematic review of PubMed was conducted to identify studies examining local anesthetic use in DR procedures. Studies were screened for relevance, full-text availability, and methodological rigor. Fourteen studies met the inclusion criteria and were evaluated for anesthetic type, dosage, application route, co-administration, and patient outcome. The Joanna Briggs Institute (JBI) critical appraisal tools were used to evaluate the methodological quality and risk of bias for each included study. Lidocaine emerged as the most frequently used anesthetic, effectively reducing pain and systolic pressure during pan-retinal photocoagulation and posterior vitrectomy. There was a statistically significant difference in the dosage of lidocaine (2.33 ± 1.00%) vs other alternative anesthetics (0.475 ± 0.05%) for surgical procedures used to treat DR. The topical application of lidocaine was preferred for its ease of administration and reduced risk of complications. Furthermore, povidone, an antiseptic agent, was frequently co-administered to disinfect the ocular surface and maintain aseptic conditions during intravitreal injections, reducing the risk of infection. Among the local anesthetics reviewed, lidocaine, administered topically or via injection, was most frequently studied and demonstrated effective procedural analgesia and favorable post-surgical outcomes. These findings suggest lidocaine is a suitable choice for DR procedures. Exploration of lidocaine's impact, consideration of patient medical history, and examination of a broader range of co-administered drugs are recommended for comprehensive insights into optimizing patient outcomes.

## Introduction and background

Diabetic retinopathy (DR), affecting an estimated 9.6 million individuals in the United States and with a prevalence rate of 26.43% among patients with diabetes in 2021, remains a leading cause of visual impairment in working-age adults [1]. DR is characterized by progressive microvascular damage to the retina, which, if left untreated, can lead to irreversible vision loss and blindness [2,3]. The disease advances from non-proliferative DR (NPDR) to proliferative DR (PDR), the latter involving neovascularization and complications such as vitreous hemorrhage or retinal detachment [4,5].

Surgical and procedural interventions, including pan-retinal photocoagulation (PRP), intravitreal anti-vascular endothelial growth factor (VEGF) injections, and pars plana vitrectomy, are commonly used to manage advanced stages of DR [6,7]. PRP works by delivering laser burns to the peripheral retina to reduce ischemia-induced neovascularization [8], while vitrectomy involves removing the vitreous gel to access and repair the retina in more severe cases [9].

Pain management during these ophthalmic procedures is essential for both patient comfort and procedural success. Local anesthesia is widely employed, with options including topical application and regional blocks such as sub-Tenon and retrobulbar injections [6,10]. Among these, local anesthetics, particularly lidocaine, are frequently chosen for their rapid onset, moderate duration of action, and favorable safety profile [10-12]. Lidocaine can be administered in several ways, including topical gel, subconjunctival injection, sub-Tenon's space injection, and retrobulbar or peribulbar blocks [13-15]. Although complications are rare, potential risks include retrobulbar hemorrhage and temporary visual disturbances [14,15].

Although both general and local anesthetics are used in ophthalmic surgery, this review focuses explicitly on the role of local anesthetic agents, with an emphasis on lidocaine, in pain control for procedures related to DR. A growing body of literature has explored alternative or adjunctive anesthetic techniques, such as the transnasal sphenopalatine ganglion block for PRP, with promising early results [12]. Individualized anesthetic care is critical given the variability in diabetic patients' pain perception, presence of autonomic dysfunction, and differing procedural needs [7,11,16,17].

Despite the widespread use of local anesthetics in retinal procedures, there is limited synthesis of the comparative effectiveness, safety profiles, and patient-reported outcomes of different agents and delivery methods. Therefore, the purpose of this review is to evaluate the current literature on the use of commonly employed local anesthetics, including lidocaine, procaine, proparacaine, bupivacaine, ropivacaine, and tetracaine. The review also briefly discusses adjunctive agents such as propofol, with a focus on their application in PRP, intravitreal injections, and vitrectomy for the treatment of DR.

## Review

Methods

A comprehensive literature review was conducted using the PubMed database to identify studies evaluating the use of anesthetic agents in the surgical treatment of DR. The search strategy was designed to capture a broad range of studies by including multiple commonly used anesthetics. Specifically, the following search phrases were entered in the title and abstract fields: "Diabetic retinopathy" AND "lidocaine" (MeSH) "Diabetic retinopathy" AND "proparacaine" (MeSH), "Diabetic retinopathy" AND "procaine" (MeSH), "Diabetic retinopathy" AND "tetracaine" (MeSH), "Diabetic retinopathy" AND "xylocaine" (MeSH), "Diabetic retinopathy" AND "bupivacaine" (MeSH), and "Diabetic Retinopathy" AND "propofol" (MeSH), "Diabetic retinopathy" AND "ropivicaine" (MeSH). No language, publication type, or date filters were applied to ensure the inclusion of all potentially relevant literature.

The initial search was then screened for duplicates, and each article was evaluated for full-text availability. The title and abstract of the remaining articles were reviewed for topical relevance and data sufficiency. The inclusion criteria were as follows: articles discussing anesthetics during procedures for DR, providing sufficient detail of the anesthetics used, and reporting data relevant to pain reduction. The exclusion criteria were lack of direct relevance to surgical anesthetic use in DR procedures, insufficient data, or a primary focus on other disease processes.

**Figure 1:**
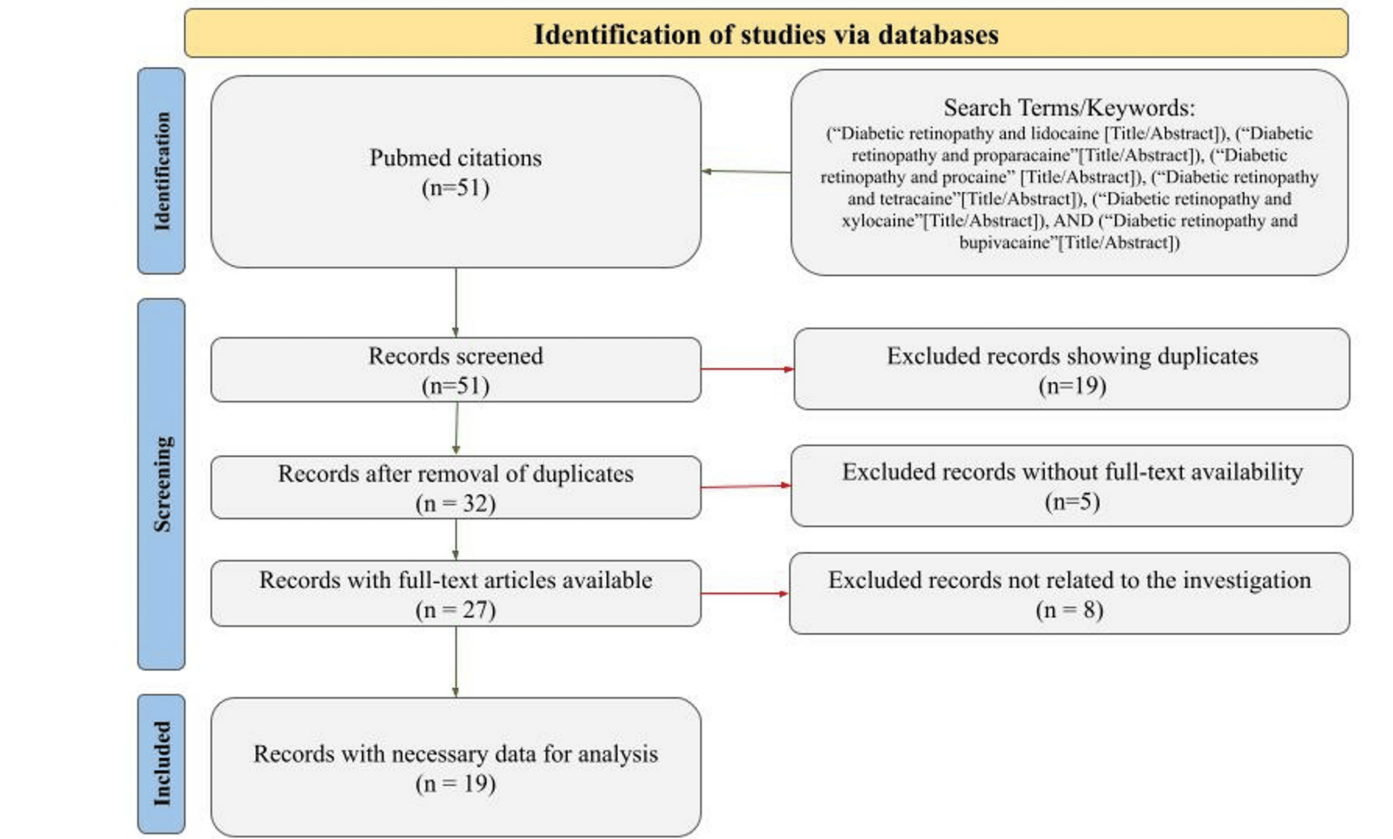
PRISMA flow diagram illustrating the selection of studies for the review on anesthesia use in diabetic retinopathy surgery. PRISMA: Preferred Reporting Items for Systematic Reviews and Meta-Analyses

The included studies were evaluated based on the type of DR procedure performed, the anesthetic agent used, dosage and concentration, route and site of administration, and reported patient outcomes such as pain control and systolic blood pressure. Co-administered agents, when applicable, were also documented. To ensure transparency in the study selection process, a Preferred Reporting Items for Systematic Reviews and Meta-Analyses (PRISMA) flow diagram was used, as shown in Figure 1.

To evaluate the methodological quality and risk of bias of the included studies, we used the Joanna Briggs Institute (JBI) Critical Appraisal Tools, selecting the appropriate tool based on study design. Specifically, we used the JBI Checklist for Analytical Cross-Sectional Studies for observational studies with comparator groups, the JBI Checklist for Case Series for descriptive, multi-patient reports without a control group, and the JBI Checklist for Case Reports for individual patient reports. Each study was independently assessed for key methodological criteria, including patient selection, validity of exposure and outcome measurement, consideration of confounding factors, statistical analysis, and clarity of reporting. Discrepancies were resolved by consensus between the two authors. Based on these assessments, studies were categorized as having low, moderate, or high risk of bias.

Results

The initial search identified a total of 51 citations. These records were first screened for duplicates, which resulted in the exclusion of 19 studies. This left 32 unique articles that underwent further evaluation. Each article was then assessed for full-text availability. Five of the 32 studies were excluded because the full text was not accessible. The remaining full-text articles were reviewed for topical relevance and data sufficiency. Eight additional studies were excluded at this stage because they did not meet the inclusion criteria. The final results of our literature review showed 19 papers that examined the effects of different anesthetics on the surgical treatment of DR (Table 1). Studies explored a wide range of anesthetics, areas of application, and co-administered anesthetics.

**Table 1:** Studies of local anesthetic use in retinal procedures for diabetic retinopathy. IOP: intraocular pressure

Author (Year)	Type of Treatment	Type of Anesthetic	Dosage	Co-administration	Area of Application	Patient Outcome/Complications	Note(s)
Tong et al. (2018) [18]	Panretinal photocoagulation	Lidocaine	2%	0.4% Oxybuprocaine	Gel (topically)	N/A	Results demonstrate that using 2% lidocaine gel to supplement topical anesthesia resulted in a more favorable pain score.
Wu et al. (2006) [19]	Panretinal photocoagulation	Lidocaine	2%	N/A	Peribulbar	Pain score of 0.8	The lidocaine-treated group (16.51±20.44 mmHg) had the least increase in systolic pressure among all the groups. Compared with the control group, the difference reached statistical significance (P=0.043).
Yepez et al (2000) [20]	Posterior vitrectomy	Lidocaine	4%	N/A	Topically (drops)	The most pain patients had was grade 1 (of 4) during most of the procedure and grade 2 (mild) during pars plana sclerotomy, external bipolar cautery, and conjunctival closure.	Posterior vitrectomy using topical anesthesia (4% lidocaine drops) was performed prospectively in 134 eyes (134 patients) with various vitreoretinal diseases, including severe proliferative diabetic retinopathy.
Kallio et al. (1999) [6]	Panretinal photocoagulation	Lidocaine	1%	N/A	Retrobulbar/peribulbar block	The overall patient outcome in the lidocaine group showed that the lidocaine effect was mainly abolished at the time of discharge, and the lidocaine group experienced a higher frequency of inadequate analgesia compared to the ropivacaine group.	
Kallio et al. (1999) [6]	Panretinal photocoagulation	Ropivacaine	0.2%	Hyaluronidase	Retrobulbar/peribulbar	76% of the ropivacaine cases were reported to have an adequate dose.	N/A
Tesha et al. (2010) [21]	Panretinal Photocoagulation	Lidocaine	2%	N/A	Subconjunctivally	In the primary study group, 59% of patients in the lidocaine group experienced pain compared with 97% in the sham treatment group.	
Sugisaka et al. (2007) [16]	Vitrectomy	Lidocaine hydrochloride	2%	Triancimilone	Retrobulbar injection	90.1% experienced visual sensations, 8.9% saw the surgeon’s hands/fingers, and 5.9% of patients were bothered by sensations.	Sensations persisted despite complete pain control.
Kaderli et al. (2006) [10]	No treatment evaluated	Lidocaine	4%	N/A	Subconjunctival and gel topically	Subconjunctival hemorrhage occurred in 32% of eyes after subconjunctival injection, whereas no anesthesia-related complication developed in the topical group.	The study focused on comparing the effectiveness of topical and subconjunctival anesthesia in intravitreal injection administrations using lidocaine.
Matharu et al. (2016) [22]	Panretinal photocoagulation	Lidocaine4 mL	mL of 4% lidocaine	Topical brimodidine/timolol, brinzolamide, travoprost	Retrobulbar injection to the right eye	The patient displayed immediate pain and no light perception following the injection. IOP elevated to 53 mmHg.	The patient had an extensive medical history. PRP was performed after failed intravitreal bevacizumab.
Stevens et al. (1993) [23]	Panretinal photocoagulation	Lidocaine (lignocaine)	2%	N/A	One-quadrant, inferior-nasal, sub-Tenon delivery of 1.5-2 mL plain 2% lignocaine	Adverse events: All 12 patients developed a subconjunctival hemorrhage occupying one or two quadrants of the bulbar conjunctiva and mild chemosis in the inferior-nasal quadrant. Two patients developed marked ocular pain with nausea and vomiting 4 hours after administration of the anesthetic and laser treatment, which settled over the same day. This coincided with the recovery of ocular sensation as the plain lignocaine 2% effect subsided. Neither patient had a rise in intraocular pressure.	The study demonstrated that sub-Tenon irrigation with 2% plain lignocaine was well tolerated and provided significantly better anesthesia than topical treatment alone, with most patients preferring this method for future PRP treatments. However, some adverse events were observed, and one patient was intolerant of PRP despite the sub-Tenon delivery.
Li et al. (2020) [24]	Vitreous retinal surgery	Propofol	N/A	Cisatracurium, rocuronium, fentanyl	Intravenous injection	Decreased IOP values	N/A
Akar et al. (2004) [25]	Photocoagulation	Lidocaine	2%	Epinephrine and bupivacaine 0.75% in equal volumes	Retrobulbar injection	Acute orbital volume change following retrobulbar injection may cause significant topographic evidence of optic disc edema lasting approximately 1 week.	
Muqit et al. (2010) [26]	Panretinal photocoagulation (20 ms and 100 ms)	Oxybuprocaine	0.4% (Topical) (5 drops over 5 minutes)	N/A	Topically (drops)	No ocular complications/adverse events	Using only topical 0.4% oxybuprocaine, with a 20ms PRP, was significantly more comfortable for patients than the traditional 100 ms PRP. The treatment was associated with decreased anxiety, pain,and photophobia compared to 100 ms.
LaHood et al. (2010) [27]	Intravitreal injection of Avastin	Lidocaine (xylocaine)	2% gel 1% injection	N/A	2% Lignocaine gel and 0.2 mL subconjunctival injection of 1% xylocaine	The overall patient outcome in the lidocaine group showed that all anesthetic techniques were effective in producing low pain scores.	In the immediate post-injection period, topical lignocaine gel appeared to be a less effective method of anesthesia when compared to the use of subconjunctival xylocaine alone or in combination with gel. There was no statistically significant difference in pain scores between the three groups 24 hours post-injection.
Oakley et al. (2018) [28]	Catheterization pain relief of IOP	Bupivacaine	3 mL 0.5% six hourly	Subconjunctival cephazolin	Sub-tenon space via catheter, sutured to the sclera. The distal end of the catheter contained a syringe for anesthesia.	Minimized pain	Pain was minimal following the dose, and 6 hours following the last anesthetic dosage.
Rifkin et al. (2012) [29]	Intravitreal injection for diabetes-related macular edema	Proparacaine HCL	0.5% HCl gel, single drop 3x (5 minutes)	Povidone (1 drop) before and after injection	Topical	Proparacine has previously shown more toxicity compared to tetracaine. Previous literature has shown better pain management, though results demonstrated tetracaine was better.	Tetracaine received the lowest pain score, followed by proparacaine, and lastly TetraVisc (most painful). The magnitude of the difference was fairly small. The most significant difference was seen between tetracaine (markedly reduced pain). Propocaine and TetraVisc were very similar, with proparacaine being slightly better.
Friberg et al. (1995) [30]	Photocoagulation with diode laser	Proparacaine HCL	0.5% HCL solution, 1 drop	N/A	Topical, retina	No outcomes relevant to proparacaine were listed.	The pain scale was compared among pulse waveforms. The anesthetic (proparacaine was consistent among all groups.
Mafrici et al. (2023) [31]	Panretinal photocoagulation	Oxybuprocaine HCl	0.4%	2% Subconjunctival lidocaine PRN	Topical	5 eyes (23.8%) receiving topical anesthesia stopped the treatment due to pain. 5 eyes (23.8%) receiving combined anesthesia had subconjunctival hemorrhage.	Patients undergoing combined anesthesia had a significantly decreased number of interruptions in a single session, with substantially lower pain perception.
Nursalim et al. (2024) [32]	Panretinal photocoagulation	Tetracaine, lidocaine	Tetracaine 0.5% eye drops, lidocaine 2% gel	Hydroxypropyl methylcellulose as a lubricant	Topical, gel	No adverse events were reported in either group. Pain scores did not show significant differences.	Tetracaine was found to be more acidic (pH of 4.54) than lidocaine (pH 6.37).

Furthermore, Table 2 summarizes the participant characteristics and overall risk of bias assessments for the included studies evaluating anesthetic methods used in ophthalmologic procedures. Among the 18 studies, seven were randomized controlled trials (RCTs) classified as low risk of bias due to apparent randomization, blinding, and standardized outcome measurement. A moderate risk of bias was assigned to eight studies, which were controlled clinical trials or observational cohorts with methodological limitations, such as unclear randomization or incomplete blinding. The participants varied across studies, including those undergoing pan-retinal photocoagulation, posterior vitrectomy, intravitreal injections, and vitreoretinal surgery. Most studies reported adequate descriptions of inclusion criteria and patient demographics.

**Table 2:** Risk of bias using the Joanna Briggs Institute (JBI) Critical Appraisal Checklists appropriate for each study design.

Author (Year)	Study Type	Overall Risk of Bias	Justification Summary
Tong et al. (2018) [18]	Controlled Clinical Trial	Moderate	Controlled design but unclear randomization and blinding details
Wu et al. (2006) [19]	Controlled Clinical Trial	Moderate	Controlled, but randomization and blinding not clearly described
Yepez et al. (2000) [20]	Prospective Cohort Study	Low	Prospective, clear inclusion, consistent outcome reporting
Kallio et al. (1999) [6]	Controlled Clinical Trial	Moderate	Controlled but randomization and blinding unclear
Tesha et al. (2010) [21]	Controlled Clinical Trial	Moderate	Sham control but limited randomization and blinding info
Sugisaka et al. (2007) [16]	Observational Case Series	Moderate to High	No control group; subjective outcomes
Kaderli et al. (2006) [10]	Cohort Study	Moderate	Non-randomized cohort; potential confounding
Matharu et al. (2016) [22]	Case Report	High	Single patient, no controls
Stevens et al. (1993) [23]	Case Series	Moderate	Small series, no control
Li et al. (2020) [24]	Observational Cohort Study	Moderate	No control, observational design
Akar et al. (2004) [25]	Observational Cohort Study	Moderate	No control group, potential confounders
Muqit et al. (2010) [26]	Randomized Controlled Trial	Low	Randomized, controlled, objective outcomes
LaHood et al. (2011) [27]	Controlled Clinical Trial	Moderate	Controlled but unclear randomization and blinding
Oakley et al. (2019) [28]	Case Series	High	Small case series, no control
Rifkin et al. (2012) [29]	Randomized Controlled Trial	Low	Proper randomization and blinding; controlled design
Friberg et al. (1995) [30]	Randomized Controlled Trial	Low	Well-designed RCT, objective pain scale use
Mafrici et al. (2023) [31]	Controlled Clinical Trial	Moderate	Controlled but randomization unclear
Nursalim et al. (2023) [32]	Randomized Controlled Trial	Low	Randomized with blinding, well-reported

Discussion

Most Common Local Anesthetic Agents

The most commonly applied anesthetic for the surgical treatment of DR appears to be lidocaine. Studies have shown that lidocaine is used in various forms, including 2% lidocaine gel topically, 2% lidocaine subconjunctivally, and 4% lidocaine topically, for procedures such as pan-retinal photocoagulation and posterior vitrectomy. Lidocaine has been favored for its effectiveness in managing pain, with studies demonstrating favorable pain scores and a reduction in systolic pressure [18]. In one study involving pan-retinal photocoagulation, lidocaine was shown to have no significant effect on intraocular pressure, in contrast to agents like propofol, which decreased intraocular pressure during vitreous retinal surgery, illustrating lidocaine's advantage in maintaining physiological stability and minimizing variables that could affect surgical outcomes [23,24]. The standard dosage of lidocaine may be attributed to its well-established efficacy and safety profile. Lidocaine is a widely used local anesthetic with a rapid onset of action and a relatively long duration of effect, making it suitable for ophthalmic procedures. The variations in lidocaine usage could be influenced by factors such as the specific procedure being performed, patient characteristics, and physician preference.

Additionally, the availability of different formulations and concentrations of lidocaine may also contribute to variations in its usage. It is important to note that, while lidocaine appears to be commonly used, the choice of anesthetic can depend on various factors, including the specific requirements of the procedure and the patient's medical history. Therefore, selecting the most appropriate anesthetic should be based on a careful assessment of individual patient needs and the nature of the surgical intervention.

Lidocaine: Analgesic and Hemodynamic Effects

Lidocaine has been shown to provide significant pain reduction in DR procedures, such as pan-retinal photocoagulation. In one double-blind study, the addition of topical 2.0% lidocaine demonstrated a significant reduction of pain surrounding a pan-retinal photocoagulation procedure [32]. In Tong et al. [18], participants were divided into two groups: Group A received 0.4% oxybuprocaine + methylcellulose gel, and Group B received 0.4% oxybuprocaine + 2.0% lidocaine gel. Patients reported their pain level (0-10 range). The study divided participants into two groups during surgery and immediately after the operation. The lidocaine Group (B) demonstrated a statistically significant pain reduction when compared to the methylcellulose group. In addition to pain reduction, lidocaine demonstrated a potential increase in the duration of the overall anesthetic effect [18]. Lidocaine is suitable, particularly in pan-retinal photocoagulation procedures, most notably in minimizing the impact on the procedure-associated blood pressure rise. In another pan-retinal photocoagulation procedure, the injection of lidocaine demonstrated a substantial decrease in patient blood pressure compared to the control in Wu et al. [19]. Those who received 2.0% lidocaine Injections saw a reduction in systolic pressure (p = 0.043). All groups experienced an increase in diastolic pressure; however, when lidocaine was administered, it minimized the increase in diastolic pressure (p = 0.030) [19].

Furthermore, the use of topical anesthesia, particularly lidocaine, has been associated with a lower risk of complications, such as hemorrhage, compared to injection-based approaches. For instance, a study by Kaderli et al. [10] compared the effectiveness of topical and subconjunctival anesthesia in intravitreal injection administrations using lidocaine and found that subconjunctival hemorrhage occurred in 32% of eyes after subconjunctival injection, whereas no anesthesia-related complications developed in the topical group [10]. Overall, the focus on topical application of lidocaine in the data suggests that this method represents a significant area of application for anesthesia in the surgical treatment of DR, offering effective pain management and a lower risk of complications such as hemorrhage compared to injection-based approaches.

Routes of Local Anesthetic Administration

Topical application of anesthetics is often preferred in the surgical treatment of DR due to its ease of use and reduced risk of complications [19]. It allows for non-invasive administration, making it especially suitable for pan-retinal photocoagulation and intravitreal injections. Regional blocks such as sub-Tenon, peribulbar, and retrobulbar techniques provide more profound analgesia and ocular akinesia in cases requiring deeper or more sustained anesthesia [25]. Additionally, local injection methods, such as subconjunctival administration, are sometimes employed to deliver anesthetic agents directly to the targeted ocular tissues, providing localized pain relief with relatively low systemic absorption. Moreover, topical anesthetic drops are commonly applied for comfort during laser surgery for DR. A local anesthetic may be administered in cases where extensive laser treatment is needed or if the patient is very sensitive [18]. In some instances, posterior vitrectomy has been performed using topical anesthesia with 4% lidocaine drops [10]. However, for more complex vitreoretinal procedures, a combination of regional anesthetic techniques with general anesthesia is typically used in the United States [20].

Co-administration Practices

Povidone is a drug commonly co-administered during the surgical treatment of DR, typically paired with tetracaine HCl ophthalmic solution during intravitreal injections for diabetic macular edema. Its use is likely due to its role in ocular surface disinfection and maintaining aseptic conditions during injections. Povidone, known as polyvinylpyrrolidone, is widely used to prevent microbial contamination in invasive ophthalmic procedures, including intravitreal injections [33]. Co-administration of povidone before and after injections helps reduce the risk of infection and ensures procedural safety [34]. It may also help maintain a clear visual field and minimize post-injection complications, aligning with standard practices in aseptic technique during DR treatments [33].

In comparison, lidocaine was not as frequently co-administered with other drugs, likely due to its proven efficacy as a standalone anesthetic. Available in gel, drop, and subconjunctival forms, lidocaine has been well studied and shown to be effective for pain control and reducing systolic pressure during procedures such as pan-retinal photocoagulation and posterior vitrectomy [26,35-37]. Its infrequent co-administration may be due to its comprehensive pain-relief capabilities [38-40]. Additionally, the selection and method of anesthetic use are often tailored to each procedure and patient, influencing whether co-administration is necessary [41,42].

Clinical Efficacy and Broader Applications of Lidocaine

The most common post-surgical outcome observed was effective pain management, which was seen in nine studies [18-21,26-31] and reduced systolic pressure, particularly with lidocaine use [19]. Lidocaine's rapid onset and versatility in form allow for tailored administration, contributing to improved comfort and outcomes [43]. Studies confirm its effectiveness in minimizing pain and reducing systolic pressure, especially during pan-retinal photocoagulation and posterior vitrectomy [19]. The efficacy of lidocaine in post-op pain control is well documented. For instance, one study on high-risk vascular surgery patients showed lidocaine infusion improved perioperative pain management [44]. An international consensus statement supports lidocaine's role in enhancing recovery and reducing post-op pain [44]. Moreover, a systematic review found that perioperative intravenous lidocaine consistently improved postoperative pain scores in abdominal surgeries [45,46]. While systemic lidocaine administration is not routinely used in ophthalmic procedures, these findings highlight lidocaine's broader analgesic efficacy. This evidence supports the potential utility of lidocaine in ophthalmic pain management, particularly when considering its strong safety profile and effectiveness across diverse surgical contexts [45]. Given the increasing prevalence of diabetic retinal disease worldwide [46], optimizing perioperative pain control in ophthalmology remains a critical and evolving area of clinical care.

These findings offer real-world guidance for ophthalmic surgeons managing DR. Data from this review highlight the effectiveness of various anesthetics, including lidocaine, buprenorphine, proparacaine, tetracaine, and procaine, in providing pain control and improving patient comfort. Lidocaine, in particular, demonstrated frequent use and consistent results in pain relief and systolic pressure reduction during standard procedures, making it a strong candidate for anesthetic selection in DR surgeries. Additionally, the review provides insight into drug co-administration practices, such as pairing povidone with tetracaine to maintain sterility and minimize complications during injections. These findings can inform physicians' decisions regarding anesthetic and co-administered drug selection to optimize patient outcomes.

## Conclusions

This review examined the use of anesthesia across 15 surgical interventions (e.g., pan-retinal photocoagulation, intravitreal injections, and posterior vitrectomy) for advanced DR. The most commonly used anesthetics were lidocaine, bupivacaine, proparacaine, tetracaine, and procaine. However, the study was limited by its narrow focus on fewer than 10 anesthetics. Future research should explore a broader range of anesthetics to validate these findings. Moreover, the patient’s medical history and procedural specifics were not fully accounted for, representing another limitation. Lidocaine was most frequently used, but should be further studied in both topical and injectable forms, especially in the context of patient history. Similarly, the range of co-administered drugs was limited, highlighting a need to investigate more combinations. Further research on surgical interventions and anesthetic strategies for DR is essential to improving patient outcomes.
